# Vampire Bats and Wild Boars in Northern Paraná: One Health Perspectives on a Novel Report

**DOI:** 10.1155/sci5/8861696

**Published:** 2025-09-15

**Authors:** João Gabriel Feriato do Nascimento, Jader Almeida de Barros Silva, Flávio Haragushiku Otomura, Marco Antonio Zanoni, Matheus Pires Rincão, Diego Resende Rodrigues

**Affiliations:** ^1^Department of Biological Sciences, State University of Northern Paraná, Bandeirantes, Paraná, Brazil; ^2^Department of General Biology, State University of Londrina, Londrina, Paraná, Brazil

## Abstract

Since its introduction to the Americas in the early 20th century, the wild boar (*Sus scrofa*) has affected Brazilian ecosystems and may have contributed to the spread of zoonotic diseases, especially rabies. Its interactions with the common vampire bat (*Desmodus rotundus*) can increase the risk of rabies transmission. These interactions remain poorly documented, particularly in the São Francisco Forest State Park, a conservation unit in the north of Paraná. In this study, we used camera traps to record three interactions between *D. rotundus* and *S. scrofa*, revealing a potential new route for zoonotic spread. Urbanization expansion and forest fragmentation further raise the risk of rabies transmission to animals and humans. Our findings highlight the need for policies and strategies to control wild boar populations and monitor vampire bats to protect public and environmental health in the region.

## 1. Introduction

The wild boar (*Sus scrofa* Linnaeus, 1758) was introduced to South America in the early 20th century. In Brazil, it was first recorded around 1990, entering from Uruguay [1]. Initially limited to the southern regions, the species quickly expanded its range [2]. Its high reproductive rate, low predation pressure, and ability to hybridize with domestic pigs (*Sus scrofa domesticus* L.), producing fertile offspring known as “wild pigs,” make it a highly invasive species [2, 3].

The wild boar and its hybrids rank among the 100 most invasive species worldwide, causing serious environmental and economic damage [4]. They destroy native vegetation and crops through intensive herbivory, rooting, and soil disturbance. They also compete with and prey on native animals and serve as reservoirs for several zoonotic pathogens [5–7].

Their invasive success is primarily driven by two key traits: a broad omnivorous diet and high adaptability to human-modified landscapes. As dietary generalists, wild boars feed on a wide range of resources, including diverse plant parts, invertebrates, small vertebrates, and carrion [8, 9]. This flexible diet matches behavioral adaptations. In anthropogenic environments, wild boars often shift to nocturnal or crepuscular activity and use forest fragments as corridors to move between plantations, likely to avoid perceived threats [10–12].

Wild boars and their hybrids carry several zoonotic pathogens (e.g., viral, bacterial, or macroparasitic) that pose serious health risks to humans. Some of these pathogens can cause chronic, difficult-to-treat, and occasionally fatal infections, including rabies (Rhabdoviridae), cryptosporidiosis (*Cryptosporidium*), giardiasis (*Giardia*), brucellosis (Brucellaceae), tuberculosis (Streptococcaceae), and leptospirosis (Leptospiraceae), among others [5]. According to Rahman et al. [13], many zoonotic diseases arise from interactions between humans and infected dogs, cats, birds, cattle, or accidental contact with disease vectors.

Wild boars and their hybrids are among the most damaging invasive species in the Brazilian Atlantic Forest [1]. This biome holds some of the world's highest levels of biodiversity and endemism [14]. However, it has been extensively degraded, with most of its remaining forest reduced to small, isolated fragments under intense human pressure [15]. In this fragmented landscape, wild boars accelerate environmental degradation by harming local fauna and flora.

Records of wild boars in the São Francisco Forest State Park (SFFSP), a conservation unit in Northern Paraná, date back to 2015. Their presence was documented in the park's management plan through interviews with staff and visitors [16]. However, from 2015 to 2024, no evidence of *S. scrofa* within the park was reported in the literature [17].

SFFSP lies within an agricultural matrix [18, 19] (Figure S1), where the presence of wild boars poses significant environmental, economic, and public health risks due to its proximity to urban areas. Livestock farming and monoculture production are also common in the region and play a key role in the local economy. Recent studies estimate that zoonotic diseases account for approximately 20% of global agricultural productivity losses [20]. In Brazil, these diseases not only reduce productivity but also risk trade embargoes on animal-derived products in both domestic and international markets [20].

The magnitude of the problem and the wide-ranging impacts of *S. scrofa* on ecosystem services make wild populations of this species a particular interest for research under the One Health approach. One Health emphasizes the inseparable integration of human, animal, and environmental (ecosystem) health, recognizing their close interdependence [21].

The presence of *S. scrofa* in SFFSP is concerning due to its interaction with the common vampire bat (*Desmodus rotundus* Geoffroy, 1810), which has been reported in several regions of Brazil and worldwide [22]. This interaction poses a risk to the One Health triad, as hematophagous bats are major reservoirs of the rabies virus (RABV) and can transmit it to wild boar, their hybrids, native wildlife, and domestic animals near the conservation unit [22–24].

In addition, wild boars and their hybrids pose a risk of disease transmission through interactions with other swine and, in some cases, through attacks on humans, events that have been well documented in Brazil [3]. Rabies is a viral zoonosis with no specific treatment; therefore, strict pre-exposure or post-exposure prophylaxis is essential [25]. This study reports three visual records captured by camera traps of common vampire bats (*D. rotundus*) feeding on wild boars (*S. scrofa*) in a seasonal semi-deciduous forest remnant of the Atlantic Forest Biome, in Northern Paraná, Southern Brazil.

## 2. Methods

SFFSP (23°09′55″S, 50°33′51″W; center of the fragment) is a conservation unit located in Northern Paraná, Brazil. It lies within a region of the Atlantic Forest where vegetation is classified as seasonal semi-deciduous [16]. According to Köppen climate classification, the region has a Cfa climate—humid subtropical mesothermal [26]—with average rainfall between 1200 and 1400 mm, distributed unevenly throughout the year [27]. The surrounding landscape is dominated by cattle pastures and monoculture crops, mainly maize (*Zea mays* L.), soybeans (*Glycine max* (L.) Merrill), sugarcane (*Saccharum officinarum* L.), and wheat (*Triticum* spp. L.) (Diego Resende Rodrigues, personal communication).

This research was conducted under the One Health Applied Research Program, Public Call 13/2019 from the Department of Sustainable Development and Tourism (SEDEST, Brazilian acronym) and Araucária Foundation (FA). The project, titled “Assessing the risk of emerging zoonoses derived from the biological invasion of *S. scrofa* to São Francisco Forest State Park,” aimed to assess whether the nonnative and invasive species of wild boar (*S. scrofa*) and the common vampire bat (*D. rotundus*) could act as reservoirs and transmitters of zoonoses within the SFFSP Conservation Unit.

This study used eight Bushnell camera traps (model Prime Low-Glow 24mp-119932C), configured to record 30-s videos for each motion event. If movement continued, the camera automatically started a new recording. The monitoring took place from October 2021 to October 2023. A total of 96 camera trap locations were established within the conservation unit using QGIS software. These points were randomly and evenly distributed, spaced 200 m apart and at least 200 m from the forest edge to reduce the risk of theft, an issue previously reported in the area.

**Figure 1:**
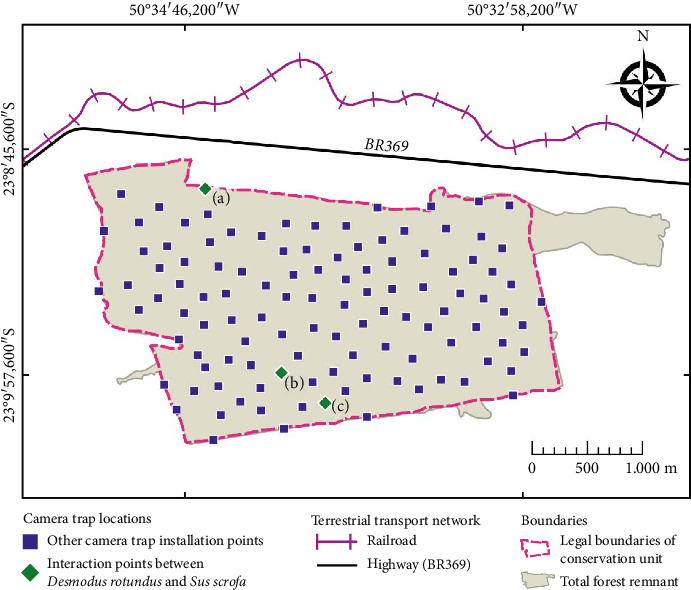
Map of camera trap locations within the São Francisco Forest State Park (SFFSP). The letters (a), (b), and (c) indicate the sites corresponding to the records shown in Figure 2.

Twelve field campaigns were carried out. In each campaign, the eight cameras were deployed for 20 days and then retrieved and repositioned to new sites. This approach resulted in 160 camera days per campaign, totaling 1920 camera days over the entire study period (Figure 1; see Pires et al. [17] for further details).

## 3. Results and Discussion

Three interactions between *D. rotundus* and *S. scrofa* were recorded, in which vampire bats attempted or successfully fed on wild pigs. These events occurred at different times and at three distinct locations in the SFFSP Conservation Unit.

**Figure 2:**
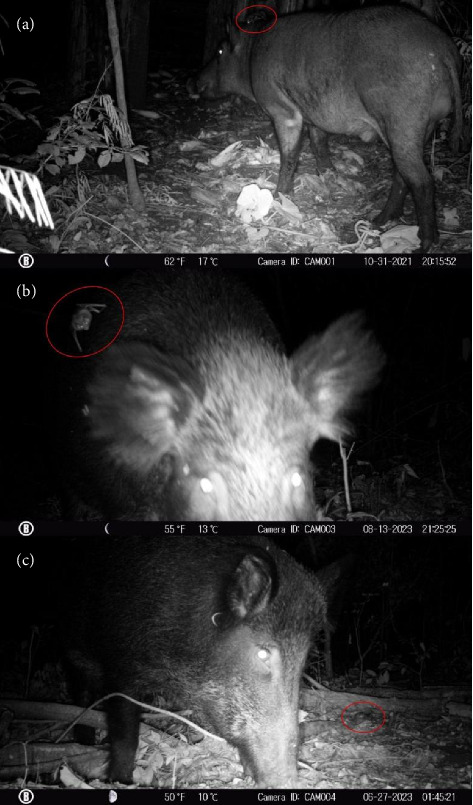
Wild boars (*Sus scrofa*) attacked by vampire bats (*Desmodus rotundus*). (a) Male wild boar, October 2021. (b) Female wild boar, August 2023. (c) Female wild boar, June 2023.

The first record, on October 31, 2021, at around 8:00 PM, captured an adult male wild boar being parasitized by a vampire bat, while other bats flew nearby as the boar fed near a camera trap (Figure 2(a)). The two subsequent records, on June 27 and August 13, 2023, at 01:54 AM and 9:25 PM, respectively, showed adult female wild boars being attacked by vampire bats at different locations within the study area (Figures 2(b) and 2(c)).

The vampire bat (*D. rotundus*) is considered a generalist predator but shows preferences for certain animal groups [22]. This preference is likely influenced by factors that facilitate feeding, such as prey with low reactivity to attacks or diurnal species that remain inactive at night when *D. rotundus* is most active.

In all records obtained during this study, the wild boars were active at the time of the vampire bat attacks. The absence of any visible reaction suggests that the wild boars were unable to detect the approach or bite of *D. rotundus*. This may be due to the bat's ability to deliver a highly precise and minimally painful incision, as previously described by Tomeček and Bodenchuk [28]. Such behavior likely facilitates successful hematophagy even when the hosts are in motion (Supporting Media S1 and Media S2).

Cattle are more accessible to *D. rotundus* because they remain in stables or pigsties at night, which can also serve as roosting sites for bats. This close proximity between prey and bat shelter likely increases the bat's feeding success, regardless of the cattle's nocturnal activity.

In a study carried out in the Atlantic Forest biome in the state of Paraná, Teider-Junior et al. [29] identified a possible relationship between wild pigs and the sylvatic rabies cycle, highlighting wild pigs as a food source for vampire bats in protected areas. Once infected with the RABV, wild boars can transmit the disease to other animals through bites or contact with carcasses contaminated with the virus. Canids, considered natural reservoirs of rabies, are among the most relevant groups for this type of transmission.

Camera traps recorded domestic dogs in the park on two occasions, chasing wild boars (Supporting Media S4 and Media S5). Although no direct evidence of wild boar hunting was found, the presence of domestic dogs poses significant risks to human and wildlife health and welfare. This concern is amplified by the presence of native canids such as *Cerdocyon thous* (Linnaeus, 1766), also found in the SFFSP [17]. In addition, potential interactions between domestic and wild animals, in both urban and natural environments, may increase threats to health and ecological balance.

Although most human rabies cases are linked to contact with the vampire bat, a significant proportion of transmissions also occur through interaction with other infected animals, both domestic and wild [13, 29]. Urban expansion, driven by population growth and real estate speculation, has brought inhabited areas closer to protected regions, increasing the likelihood of interaction between wildlife and humans [30].

Video recordings of vampire bats, RABV vectors, attacking wild pigs, combined with the dynamics of wild boar hunting, meat preparation and consumption, and ongoing monitoring and research activities in the region, indicate the presence of a potential risk group for the emergence of rabies cases. Recent studies, such as those by Teider-Junior et al. [29] and Kmetiuk et al. [24], corroborate this observation by reporting similar scenarios involving wild boar management, rabies transmission dynamics, and the formation of risk groups due to contact with this exotic invasive species.

## 4. Conclusion

Records of interactions between *S. scrofa* and *D. rotundus* in SFFSP highlight the public and environmental health risks posed by the spread of invasive exotic species in protected areas. While such interactions have been documented in the literature, their occurrence in SFFSP is novel and emphasizes the need for targeted management actions. These include wild boar population control, rabies vaccination campaigns for domestic animals, and continuous monitoring of RABV vectors. However, structural and human resource limitations within conservation units compromise the effectiveness of these initiatives, highlighting the need for targeted investments. Our findings also support the development of integrated public policies and dedicated funding for invasive species control. Further research is needed to investigate the relationship among landscape changes, wild boar dynamics, and RABV circulation, to support mitigation strategies aligned with the One Health approach.

## Data Availability

The data that support the findings of this study are openly available in Figshare at https://www.doi.org/10.6084/m9.figshare.28137392.

## Supporting Information

The data supporting the conclusions of this study, and the Supporting Information listed below, are available on Figshare at https://www.doi.org/10.6084/m9.figshare.28137392.

Supporting Information 1 Figure S1. Land use and land cover surrounding the study area.

Supporting Information 2 Media S1. Adult male wild boar parasitized by a vampire bat while feeding near a camera trap. Additional bats are seen flying nearby.

Supporting Information 3 Media S2. Adult female wild boar attacked by a vampire bat.

Supporting Information 4 Media S3. Vampire bat waiting for an opportunity before launching an attack on an adult female wild boar.

Supporting Information 5 Media S4. Sequence showing wild boars possibly being chased by dogs (May 2022).

Supporting Information 6 Media S5. Sequence showing wild boars possibly being chased by dogs (December 2022).
