# Supplementary material for: Vampire Bats and Wild Boars in Northern Paraná: One Health Perspectives on a Novel Report
Source: Scientifica (Cairo). 2025 Sep 15;2025:8861696. doi: 10.1155/sci5/8861696 (PMC12453907; doi:10.1155/sci5/8861696)
Supplement: Supporting Information 1 — Figure S1. Land use and land cover surrounding the study area. [file 8861696.f1.pdf]

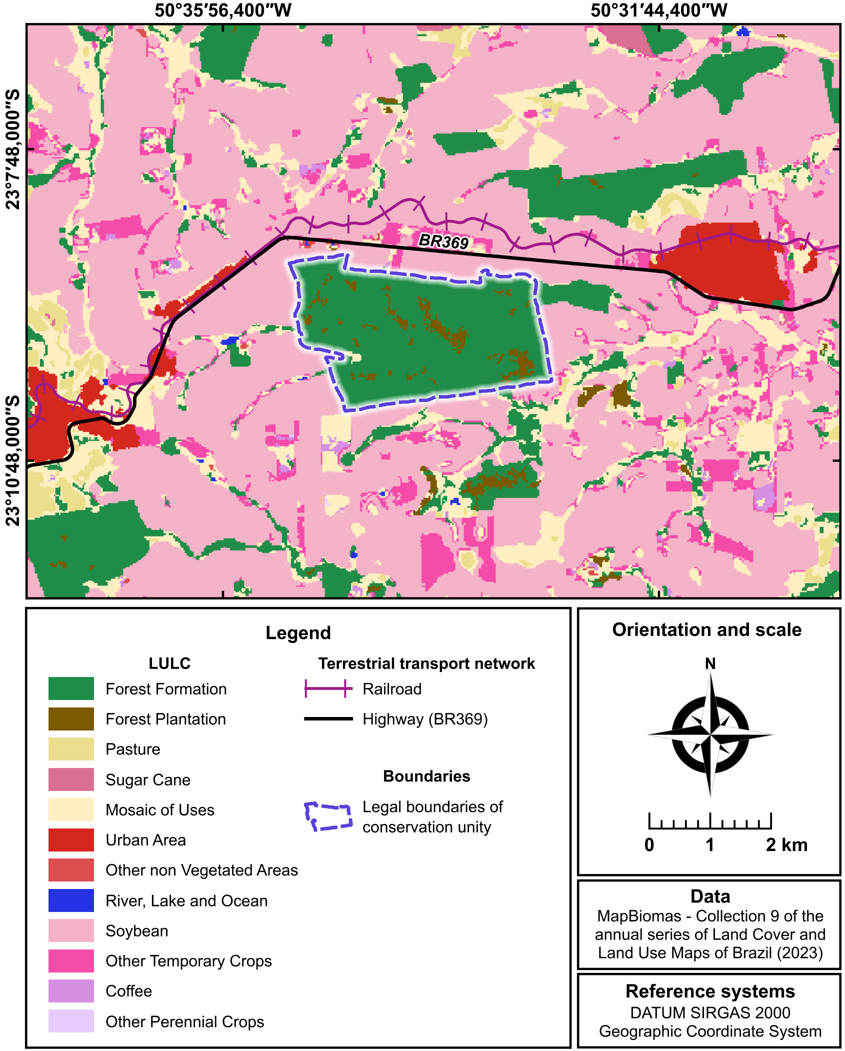
50°35′56,400″W
50°31′44,400″W
23°7′48,000″S
23°10′48,000″S
BR369
Legend
LULC
Forest Formation
Forest Plantation
Pasture
Sugar Cane
Mosaic of Uses
Urban Area
Other non Vegetated Areas
River, Lake and Ocean
Soybean
Other Temporary Crops
Coffee
Other Perennial Crops
Terrestrial transport network
Railroad
Highway (BR369)
Boundaries
Legal boundaries of conservation unity
Orientation and scale
N
0
1
2 km
Data
MapBiomas - Collection 9 of the annual series of Land Cover and Land Use Maps of Brazil (2023)
Reference systems
DATUM SIRGAS 2000
Geographic Coordinate System
